# Metabarcoding of zooplankton communities of Dianchi Lake based on the mitochondrial cytochrome oxidase subunit 1 gene

**DOI:** 10.3389/fmicb.2023.1291632

**Published:** 2024-01-24

**Authors:** Fu Cen, Shan Xu, Genshen Yin, Minghua Dong

**Affiliations:** ^1^School of Agriculture and Biotechnology, Kunming University, Kunming, Yunnan, China; ^2^Kunming Key Laboratory of Hydro-Ecology Restoration of Dianchi Lake, Kunming University, Kunming, Yunnan, China

**Keywords:** Dianchi Lake, COI gene marker, zooplankton, diversity, environmental factors correlation

## Abstract

Freshwater lakes as an essential component of the ecosystem, provide ecological resources in addition to economic source for humans. Under recent climate change scenario, preserving the biodiversity of freshwater ecosystems is crucial. This study aimed to characterize the diversity of zooplankton communities in Dianchi Lake, located in Kunming Municipality, Yunnan Province, China, using Illumina high-throughput sequencing of the cytochrome oxidase subunit 1 (COI) gene marker. A total of 18 water samples were collected including 16 from the outer sea area of Dianchi Lake: 4 from the east (E1-4), 4 from the west (W1-4), 4 from the south (S1-4), and 4 from the north (N1-4), and: 2 from the Caohai area (C1-2) as research sites. All environmental parameters including pH, ammonium (NH^4+^), total nitrogen (TN), total phosphorus (TP), chlorophyll *a* content (CHLA) were found to be insignificant (*p* > 0.05), except for chemical oxygen demand (COD) and transparency (T), which were found to be significant (*p* < 0.05). Alpha diversity indices including ACE, Chao1, Shannon, and Simpson showed non-significant differences (*p* > 0.05), indicating no variation in the richness of zooplankton communities at different locations of Dianchi Lake. However, principal coordinate analysis (PCoA) showed that most of the samples from East, West, and South groups were close to each other, showing more similarities among them, while Caohai and North group samples were distant from each other, showing more differences with other groups. Rotifera, Arthropoda, and Chordata were the top three phyla, while Keratella, Macrothrix, and Brachionus were the dominant genera. Mantel test analysis showed that COD and transparency were important environmental factors that shaped the Rotifera community structure of Dianchi Lake. In conclusion, this study provides insights on conserving the diversity of zooplankton communities in Dianchi Lake, especially by controlling COD and maintaining water transparency, in order to preserve its ecological resources and economic significance.

## Introduction

Freshwater lakes as an indispensable part of the ecosystem, provide ecological resources in addition to economic support for social civilization (Wang J.-H. et al., 2020). In the 21st century, it is crucial to preserve the biodiversity of freshwater ecosystems. The most threatened ecosystems are freshwater ecosystems, which have the highest concentration of species of any ecosystem on the planet (Dudgeon et al., 2006; Vaughn, 2010). These ecosystems are vulnerable to anthropogenic activities such as urbanization, industrialization, eutrophication, climate change, and the influx of non-native species, which have contributed to a long-term decline in biodiversity (Vörösmarty et al., 2010; Ding et al., 2015). In recent years, the biodiversity of these ecosystems has declined more dramatically than that of marine or terrestrial ecosystems (Zhao et al., 2021). Therefore, regular monitoring of freshwater ecosystems is a top priority to conserve their biodiversity. However, a thorough understanding of biodiversity is of paramount importance to protect these ecosystems.

Dianchi Lake is located in southwestern China (24°23′-26°22′N, 102°10′-103°40′E) adjacent to Kunming City, Yunnan Province, with an area of about 300 km^2^ and a depth of 4.7 m. It was considered as one of the most important reservoirs for supplying domestic water to 6.8 million residents (Wang et al., 2012; Wang Y. et al., 2018). Over the past 30 years, the lake had been increasingly polluted due to the rapid population growth in the area, the decrease in the biomass of aquatic plants and benthic animals, the increase in the biomass of phytoplankton, and the effects of discharging huge amounts of municipal and industrial wastewater into the water body (Wang et al., 2010; Li et al., 2014; He et al., 2021). A great deal of effort, including labor, material, and financial resources, has been devoted to cleaning up pollution in Dianchi Lake. The focus has shifted from conservative water quality improvement to overall ecosystem function as water quality has improved significantly (He et al., 2021).

In lake and river ecosystems, plankton diversity has been frequently used as aquatic bioindicator and considered as a crucial monitoring metric in management programs (Mimouni et al., 2015; Pineda et al., 2019). Moreover, zooplankton communities undergo seasonal and spatial variations in response to abiotic variables such as variations in water temperature and biological factors, thereby displaying distinct seasonal and spatial change patterns (Walkusz et al., 2009; Abe et al., 2020). Detecting patterns of species diversity of marine zooplankton is crucial as they are key characteristics of marine ecosystems that determine their function, sustainability, and responses to environmental variability and anthropogenic events, including climate change (Sherman et al., 2002; Friedland et al., 2020).

The traditional taxonomic approaches based on morphological features used to characterize zooplankton communities are insufficient and laborious for substantial biodiversity research, despite the fact that zooplankton possesses significant biological and economic impact in freshwater ecosystems (Deiner et al., 2015; Trebitz et al., 2017; Lee et al., 2018). With the advancements in molecular techniques, advanced metagenomics and environmental DNA (eDNA) metabarcoding have been emerged as a fast and precise approaches for studying the plankton diversity in aquatic ecosystems (He et al., 2021). This is achieved by detecting eDNA that is released into the environment from aquatic animals and plants (Fabrin et al., 2020; Jo et al., 2020). The analysis of the biological community and its relative abundance using eDNA metabarcoding technology, which combines high-throughput sequencing (HTS) with DNA sequences from various environmental samples, is widely employed in freshwater ecosystem’s protection (Lv et al., 2023). The eDNA metabarcoding approach offers comprehensive depiction about zooplankton biodiversity and help to conserve biodiversity (Xiong et al., 2020). In addition, it has been successfully employed to monitor seasonal changes in the zooplankton population (Mwagona et al., 2018; Berry et al., 2019), and also provides systematic support for investigating the aquatic environmental factors affecting zooplankton diversity (Yang et al., 2017). The COI marker has been widely used for genotyping zooplankton species due to its effectiveness in species detection and identification (Bucklin et al., 2021, 2022). Several studies have demonstrated the suitability of COI markers for zooplankton DNA metabarcoding and the advantages of using a dual COI marker for marine zooplankton (Schroeder et al., 2021). The widespread use of COI for species identification is the availability of taxonomically complete COI reference databases for marine zooplankton, as it can effectively detect at least major species (Porter and Hajibabaei, 2018; Zhao et al., 2021).

In the present study, the Illumina HTS of COI gene was performed to analyze the diversity and community structure of zooplanktons in the Dianchi Lake water and their relationship with environmental factors was investigated.

## Materials and methods

### Sampling sites and data collection

Dianchi Lake (24° 40′-25°02′N, 102°36′-102°47′E) is located in downstream of the main urban area of Kunming City, Yunnan Province. The lake is approximately 40 km long from north to south, with an average east-west width of about 7 km. The lake covers an area of 298.4 km^2^. The sea ridge in the northern part of the lake divides Dianchi Lake into two parts: Caohai and Outer Sea. Based on the previous research on the distribution of submerged plants in Dianchi Lake, a total of 18 sampling points were selected, 16 from the outer sea area of Dianchi Lake, including 4 in the east (E1-4), 4 in the west (W1-4), 4 in the south (S1-4), and 4 in the north (N1-4), and 2 from the Caohai area (C1-2) as the research points. A completely randomized design (CRD) was used with five treatments viz. one treatment from the Caohai area (C): C1 and C2; and four treatments for the Outer Sea area including the northern part of Dianchi Lake (N): N1, N2, N3, and N4; southern part of Dianchi Lake (S): S1, S2, S3, and S4; east part of Dianchi Lake (E): E1, E2, E3, and E4; and west part of Dianchi Lake (W): W1, W2, W3, and W4. A total of 30 L of water samples were collected from the upper, middle, and lower layers of each location. After thorough mixing, water samples (5 L) were taken, filtered, and concentrated to 100 ml using No. 25 planktonic net. Then these samples were loaded into sampling bottles and brought to the laboratory. Then samples were passed through a 0.45 μm filter membrane, placed in a storage tube, and stored at −80°C.

### Determination of water characteristics of Dianchi Lake

The environmental parameters of the collected water samples were determined according to the “Water and Wastewater Monitoring and Analysis Method” (4th edition) (State Environmental Protection Administration, 2002). Briefly, the pH of the water samples was determined using the pH meter; NH^4+^ concentration was measured through spectrophotometer; the total nitrogen (TN) concentration was determined using alkaline potassium persulfate oxidation ultraviolet spectrophotometry; total phosphorus (TP) was determined by ammonium molybdate spectrophotometry; the DO was measured using the standard iodometric method; the chemical oxygen demand (COD) was determined by the potassium dichromate method. The concentration of chlorophyll *a* content (CHLA) was determined using ethanol and spectrophotometry. Transparency was measured using a transparency disc.

### Genomic DNA extraction and library preparation

The genomic DNA was extracted using the E.Z.N.A.™ Water DNA kit (OMEGA Bio-Tek Inc., Doraville, GA, USA) from 18 water filter membrane samples. DNA integrity was detected by 1% agarose gel, and the concentration of DNA samples was measured by Qubit (Thermo Fisher Scientific, USA). Using the extracted genomic DNA as a template, the mitochondrial cytochrome oxidase subunit 1 (COI) gene was amplified as a barcode for zooplankton DNA. The primers mlCOIintF (5′-GGWACWGGWTGAACWGTWTAYCCYCC-3′) (Leray et al., 2013) and jgHCO2198 (5′-TAIACYTCIGGRTGICCRAARAAYCA-3′) (Geller et al., 2013) were used to amplify the target fragments with a length of about 313 kb. The PCR reaction conditions used included: 94°C, 3 min → (94°C, 30 s → 45°C, 20 s → 65°C, 30 s) 5 → (94°C, 20 s → 55°C, 20 s → 72°C, 30 s) 20 → 72°C, 5 min → 10°C, ∞. PCR products were detected by 1% agarose gel electrophoresis and then recycled by cutting the gel and subjecting it to Tris_HCl elution and 2% agarose electrophoresis using an AxyPrepDNA gel extraction kit (Axygen Biosciences, USA). Following purification, an amplicon library was formed using a standardized process for qualified PCR products, and a MiSeq Reagent Kit v2 (500 cycle) was used to conduct HTS on the Illumina MiSeq platform (Allwegene, China).

### Bioinformatics analysis

Trimmomatic with a 50 bp window size was used for the initial quality control step (Bolger et al., 2014). Bases having a Phred quality score of less than 20 were trimmed. Cutadapt was used to delete the primer sequence (Martin, 2011). FLASH (Magoč and Salzberg, 2011) was used to combine the raw paired-end reads, requiring a minimum overlap sequence length of at least 10 bp and a maximum permitted mismatch ratio of 0.1. Chimeric sequences were identified and eliminated using UCHIME (Edgar et al., 2011). The UPARSE process (Edgar, 2013) was used for OTU clustering using a 97% similarity cut-off. The OTU annotation was carried out against the BOLD reference (COI) database (Ratnasingham and Hebert, 2007). The OTU was given a higher taxonomic rank if a species attribution was not possible. All bacteria, fungi, and Viridiplantae were eliminated from the OTU annotation tables in order to concentrate solely on zooplankton in this study. Statistical analyses were performed by using the “vegan” package in R v.3.6.3 (Oksanen et al., 2019). Zooplankton communities’ saturation was analyzed using rarefaction and rank abundance curves. Venn diagram was used to display the number of various taxa that could be retrieved using COI markers at various taxonomic levels. Beta diversity analysis principal coordinate analysis (PCoA) and alpha diversity analyses (OTUs, ACE, Shannon, Simpson, and Chao1) were carried out using the Qiime software (Version 1.9.1), and the outcomes were further analyzed with R software (Version 2.15.3). An analysis of similarity (ANOSIM) was also used for further evaluation based on Bray–Curtis dissimilarity index. According to the characteristics of the measured water environmental factor data, the relationship between the variables was analyzed using Spearman’s correlation analysis with Bonferroni’s correction to account for multiple testing. To investigate the water environmental factors that drive seasonal changes in the zooplankton community, the OTU data matrix was associated to the water environmental factor matrix by using the partial Mantel test.

## Results

### Water quality parameters of Dianchi Lake

The quality parameters of Dianchi Lake water samples collected from different locations were measured and are listed in Table 1. All parameters were found non-significant (*p* > 0.05) difference among different locations except for COD and transparency which significantly (*p* < 0.05) differed. The highest COD concentration (39.00 ± 10.0 mg/L) was observed at the Caohai location, whereas the lowest concentration (12.25 ± 1.25 mg/L) was at the North point of the Outer Sea part. The highest value (65.50 ± 1.19 cm) for T was observed at the South point of the Outer Sea part, whereas the lowest value (29.5 ± 4.50 cm) was observed at the Caohai part of Dianchi Lake.

**TABLE 1:** Dianchi Lake water environmental parameters at different locations.

Sample	CHLA (μ g/L)	COD (mg/L)	DO (mg/L)	NH4^+^ (mg/L)	pH	T (cm)	TN (mg/L)	TP (mg/L)
Caohai (C)	41.50 ± 31.50	39.00^a^ ± 10.0	3.65 ± 0.25	2.75 ± 2.57	7.30 ± 0.10	29.5^d^ ± 4.50	4.90 ± 2.21	0.42 ± 0.14
East (E)	30.25 ± 12.17	13.25^b^ ± 0.63	4.23 ± 0.16	0.81 ± 0.25	7.25 ± 0.07	56.25^b^ ± 1.11	2.23 ± 0.69	0.34 ± 0.12
West (W)	31.50 ± 1.56	15.50^b^ ± 2.78	4.28 ± 0.29	0.31 ± 0.07	7.28 ± 0.05	61.50^ab^ ± 1.0	1.67 ± 0.33	0.23 ± 0.04
North (N)	14.25 ± 0.75	12.25^b^ ± 1.25	4.68 ± 0.19	0.40 ± 0.26	7.40 ± 0.04	43.25^c^ ± 1.75	2.67 ± 0.45	0.18 ± 0.03
South (S)	33.50 ± 7.12	23.25^b^ ± 3.01	4.58 ± 0.23	0.52 ± 0.16	7.35 ± 0.07	65.50^a^ ± 1.19	3.49 ± 1.20	0.16 ± 0.02

Values in the same column with different superscript letters differ significantly at *p* < 0.05. CHLA, chlorophyll *a* content; COD, chemical oxygen demand; DO, dissolved oxygen; NH4^+^, ammonia; T, transparency; TN, total nitrogen; TP, total phosphorus.

### OTUs annotation and alpha diversity analysis

**FIGURE 1:**
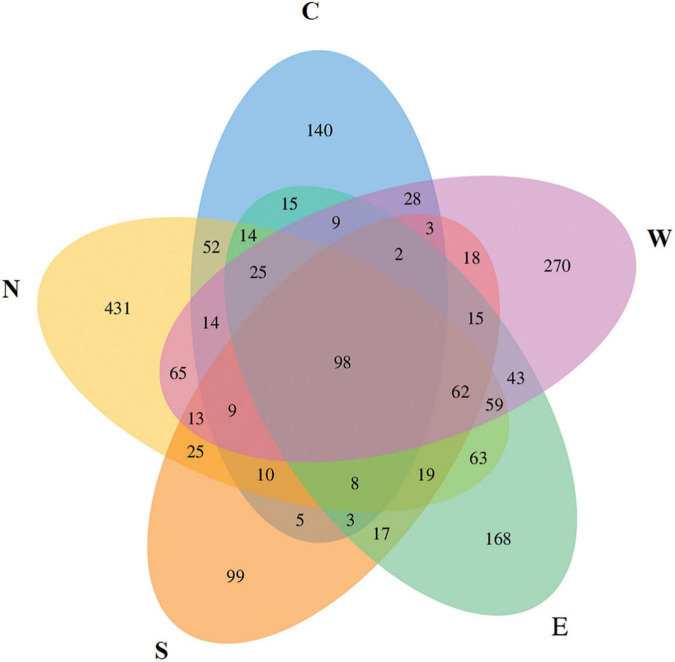
Venn diagram showing the unique and common OTUs among five group samples (East, West, North, South, and Caohai) of zooplankton communities of Dianchi Lake.

**FIGURE 2:**
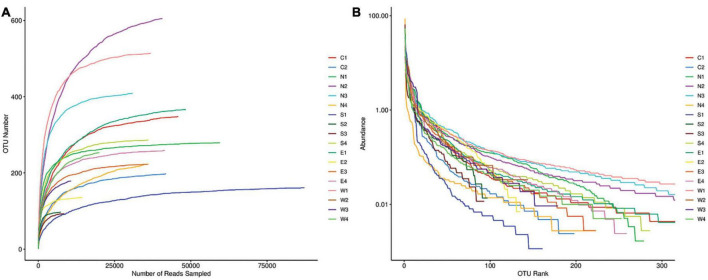
**(A)** Rarefaction and **(B)** rank abundance curves of five group samples (East, West, North, South, and Caohai) of zooplankton communities of Dianchi Lake.

We performed metabarcoding of a total of 18 samples, 4 from each of the East, West, South, and North points of the Outer Sea part of Dianchi Lake and 2 from the Caohai part of Dianchi Lake using the COI marker gene targeting the zooplankton communities. All five groups (East, West, North, South, and Caohai) shared a total of 98 OTUs among themselves, while 168, 270, 431, 99, and 140 unique OTUs were found among them, respectively (Figure 1). The rarefaction and rank abundance curves of 18 zooplankton samples stabilized with sequencing depth (Figures 2A, B). This indicated that most of the sampling locations were successfully sampled and that the data were likely saturated.

The alpha diversity analysis of the zooplankton communities of Dianchi Lake at different locations was performed using the alpha diversity indices OTUs, ACE, Chao1, Shannon, and Simpson (Table 2). All the diversity indices showed non-significant (*p* > 0.05) differences, indicating no variation in the diversity of zooplankton communities at different locations of Dianchi Lake. However, values of OTUs, ACE, and Chao1 showed the highest diversity of zooplankton communities at the northern point of the outer sea part and the lowest at the southern point of the outer sea part. Whereas, Shannon and Simpson’s indices showed the highest diversity of zooplankton communities at the East and South points of the outer sea part, respectively, and the lowest at the South and East points, respectively.

**TABLE 2:** Alpha diversity indices of zooplanktons of Dianchi Lake water samples at different locations.

Sample	OTUs	ACE	Chao1	Shannon	Simpson
Caohai (C)	273.00 ± 75.00	284.35 ± 76.75	283.45 ± 78.65	2.85 ± 0.65	0.20 ± 0.12
East (E)	245.75 ± 47.80	253.68 ± 50.67	253.52 ± 51.00	3.48 ± 0.11	0.08 ± 0.02
West (W)	223.25 ± 38.64	292.75 ± 78.30	292.18 ± 79.07	3.43 ± 0.41	0.11 ± 0.06
North (N)	379.00 ± 84.81	396.50 ± 83.76	402.65 ± 79.00	3.38 ± 0.83	0.22 ± 0.18
South (S)	159.25 ± 45.03	164.07 ± 45.03	163.90 ± 44.86	2.48 ± 0.36	0.25 ± 0.07

### Relative abundance of zooplankton communities of Dianchi Lake

**FIGURE 3:**
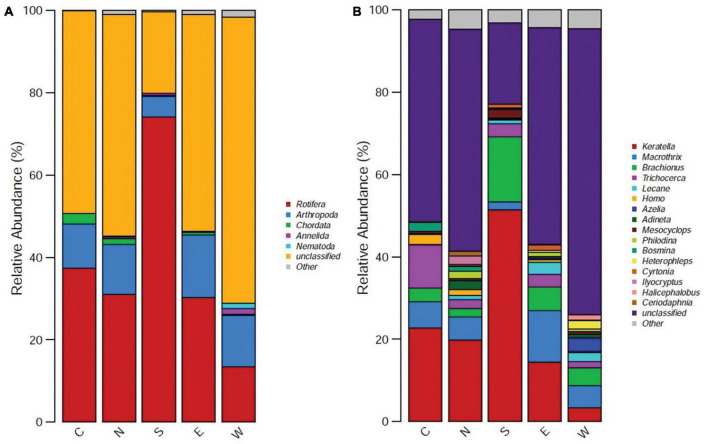
Relative abundance of five group samples (East, West, North, South, and Caohai) of zooplankton communities of Dianchi Lake at **(A)** phylum and **(B)** genus level.

The relative abundance of zooplankton communities of Dianchi Lake at different locations was analyzed at the phylum and genus level using the COI marker gene (Figures 3A, B). At the phylum level, mainly Rotifera, Arthropoda, Chordata, Annelida, and Nematoda communities were observed (Figure 3A). Among these phyla, Rotifera, Arthropoda, and Chordata were the top three phyla. The relative abundance of Rotifera was highest (74.1%) at the southern point of the Outer Sea part and lowest (13.4%) at the West point of the Outer Sea part. The relative abundance of Arthropoda was highest (15.2%) at the East point of the Outer Sea part and lowest (5%) at the South point of the Outer Sea part. Phylum Chordata communities were highly abundant (3%) at Caohai part of Dianchi Lake and the lowest (0.2%) at the southern point of the outer sea part. Keratella, Macrothrix, and Brachionus were the top three genera (Figure 3B). The relative abundance of Keratella was highest at the southern point (50%) of the Outer Sea part and lowest (4%) at the western point of the Outer Sea part. However, relative abundance of Macrothrix was highest (13%) at the eastern point of the Outer Sea part and lowest (6%) at the western point of the Outer Sea part. Communities belonging to the genus Brachionus were highly abundant (16%) at the southern point of the Outer Sea part and lowest (1%) at the northern point of the Outer Sea part.

### Beta diversity analysis

**FIGURE 4:**
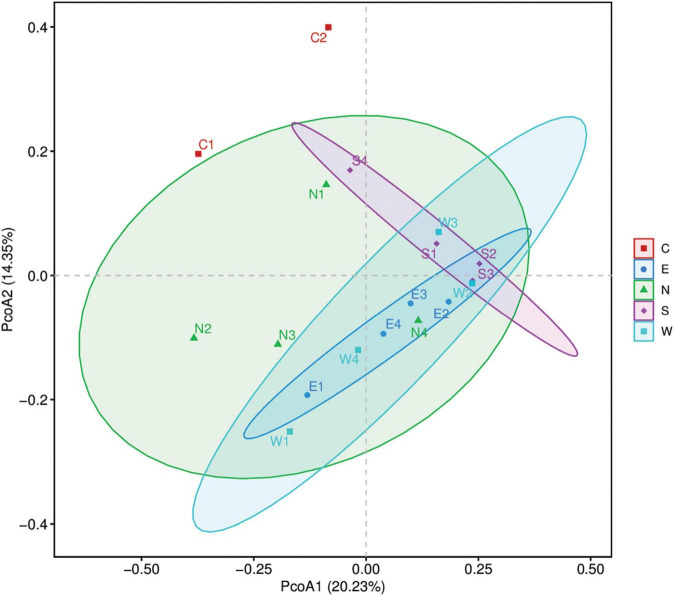
Principal coordinate analysis (PCoA) showing the similarities and differences between and within five group samples (East, West, North, South, and Caohai) of zooplankton communities of Dianchi Lake.

Principal coordinate analysis was performed to find the similarities and differences of zooplankton communities between and within the five group samples (East, West, North, South, and Caohai) of Dianchi Lake (Figure 4). The PCoA1 revealed a 20.23% of total variance, while PCoA2 revealed a 14.35% of total variance. Most of the East, West, and South group samples were close to each other showing more similarities among them, whereas Caohai and North group samples were distant apart showing more differences with other groups. Further, the ANOSIM showed the groups wise comparisons based on Bray–Curtis dissimilarity index (Table 3). The results revealed N vs. E, and E vs. W had significant (*p* > 0.05) differences between them, whereas S vs. W had more differences within the group (*R* = −0.02) compared to other groups.

**TABLE 3:** Analysis of similarity results between five group samples (East, West, North, South, and Caohai) of zooplankton communities of Dianchi Lake.

Comparison	Sample size	Group size	ANOSIM statistic *R*	*p*-Value	Permutations
C vs. N	6	2	0.25	0.33	719
C vs. S	6	2	0.32	0.2	719
C vs. E	6	2	0.89	0.07	719
C vs. W	6	2	0.64	0.07	719
N vs. S	8	2	0.15	0.21	999
N vs. E	8	2	0.35	0.05	999
N vs. W	8	2	0.03	0.41	999
S vs. E	8	2	0.22	0.14	999
S vs. W	8	2	−0.02	0.47	999
E vs. W	8	2	0.29	0.05	999
Between	18	5	0.21	0.03	999

### Environmental factors correlation

**FIGURE 5:**
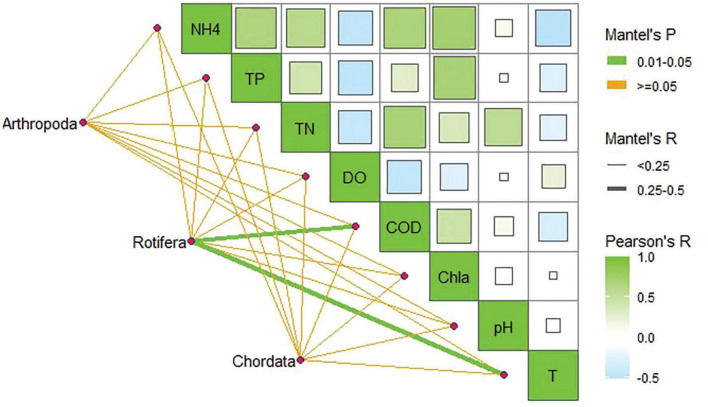
Pearson’s correlation between the top three observed phyla of zooplankton communities and water quality parameters of Dianchi Lake.

The partial (geographic distance-corrected) Mantel’s test was conducted to associate the DNA metabarcoding datasets with the distance matrix of water quality parameters based on Euclidian distances in order to better understand the environmental factors that influence fluctuations in zooplankton diversity. The findings demonstrated that the primary water quality parameters including COD and transparency significantly (*p* < 0.05) affected the Rotifera communities of Dianchi Lake (Figure 5). Further, a high positive Pearson’s correlation was observed among NH4^+^, TN, TP, COD, and CHLA parameters.

## Discussion

Zooplankton are an essential component of both marine and freshwater ecosystems, comprising some of the most abundant and diverse populations of organisms found in aquatic environments (Djurhuus et al., 2018; Xiong et al., 2019). Ecology and conservation biology continue to place a strong emphasis on understanding zooplankton diversity (Carugati et al., 2015). These studies are particularly important for aquatic ecosystems because of the complex biotic interactions and environmental drivers of their ecological processes (Steinberg and Landry, 2017; Cicala et al., 2022). Zooplankton are extremely vulnerable to environmental changes due to their small size and low ability to self-dispersal (Yang and Zhang, 2020). As a result, they are useful bioindicators for determining the health of marine ecosystems. The geographic and temporal abundance and taxonomic composition of zooplankton communities are strongly influenced by both environmental changes and biological processes (Cicala et al., 2022). These changes can negatively affect zooplankton biodiversity, which could have an impact on ecosystem services that could have economic implications (Everaert et al., 2018). In the present study, all observed environmental parameters were found to be insignificant except COD and water transparency, which differed significantly between the Outer Sea (east, west, north, and south) and Caohai parts of Dianchi Lake. COD is an important indicator of water pollution levels and can be used to assess water quality for use by living organisms (Qiong et al., 2009). A previous study showed that variations in COD can significantly alter zooplankton diversity (Zhao et al., 2021). The highest COD was observed in the Caohai part of Dianchi Lake, with the lowest transparency values, indicating more water pollution than the other locations. Dianchi Lake receives water from 29 streams. Previously, Caohai (3% of the total area) had more significant pollution problems as it received the highest pollutant load from industry and agriculture (Li et al., 2007). More recently, Caohai’s contribution to the pollution load was mainly due to the large-scale urban development taking place there (Wang et al., 2022). Many polluting facilities in the Caohai area have been closed down, the industrial layout and structure have been optimized, and the industrial pollution sources have been monitored in an effort to reduce the high pollution rates in the area. As a result, the percentage of point source pollution load has been reduced (Wang et al., 2022).

Metabarcoding of zooplankton communities of water samples from Dianchi Lake from the Outer Sea part (east, west, north, and south) and Caohai part was performed using the COI gene marker. The alpha diversity indices (OTUs, Ace, Chao1, Shannon, and Simpson) were found to be insignificant, indicating no significant variation in the richness of zooplankton communities at all sites. The highest OTUs were observed at the North site, which could be related to the low COD levels indicating low pollution, resulting in higher richness of zooplankton communities as described previously (Zhao et al., 2021). Furthermore, the beta diversity analysis PCoA showed that the Caohai part and the North point of the Outer Sea part were separated from each other, showing the significant differences of zooplankton communities with other location samples. Previous studies suggested that the spatial variations in zooplankton communities could be attributed to environmental variations (Feng et al., 2020; Wang W. et al., 2020). Our study was also consistent with these studies, as the Caohai part had the highest COD and the North Point had the lowest COD, so these locations had significant differences in zooplankton communities and were separated from other groups. Furthermore, taxonomic assignment showed that the three most abundant phyla were Rotifera, Arthropoda, and Chordata, and their relative abundance varied among groups. A 50-year regime shift in Dianchi Lake from 1957 to 2007 revealed that the biomass of Rotifera increased more than 20-fold from 1957 to 1982, which was very high compared to other groups (Wang J. et al., 2018). Due to their small size and short life cycle, rotifers exhibit increased levels of both richness and relative abundance. In addition, they exhibit resistance to a variety of environmental factors, making Rotifers the dominant species in the reservoir habitat (Nogueira, 2001). Arthropods play a crucial role in maintaining the health of ecosystems by acting as both herbivores and carnivores, consuming autotrophs, heterotrophs, and particulate organic matter, and are very sensitive to environmental changes (Fenoglio et al., 2020). In our study, the relative abundance of arthropods did not vary significantly among the different locations and was lowest at the southern point of the Outer Sea part. The phylum Keratella, Macrothrix, and Brachionus were the top three genera. Keratella and Brachionus belong to the phylum Rotifera, while Macrothrix belongs to the phylum Arthropoda. Keratella is cosmopolitan and is an indicator of the trophic state of water bodies (Haberman and Haldna, 2014). *Brachionus* spp. especially *Brachionus plicatilis* is a useful model to determine the impact of toxicity on aquatic ecosystems (Kostopoulou et al., 2012). It is preferred for these studies due to its widespread distribution, ease of culture, suitable size, rapid reproduction, and complex life cycle. *Macrothrix* spp. efficiently regulates phytoplankton biomass, mainly consuming chlorophytes and filamentous and colonial cyanobacteria, and could be used for biomanipulation in tropical eutrophic aquatic habitats (Amorim et al., 2019). Furthermore, the top three phyla Rotifera, Arthropoda, and Chordata were analyzed for correlation with environmental factors. The Mantel test revealed that Rotifera communities were driven by COD and transparency parameters, and positive correlation. However, the previous study investigated the negative correlation between COD and Rotifers (Sarang and Manoj, 2021). The difference in the correlation between COD and Rotifera phylum in our study compared to previous research may be attributed to differences in sampling methods, environmental conditions, and Rotifera species composition. Our findings revealed that zooplankton communities in Dianchi Lake were affected by environmental factors, especially COD and water transparency. The three most prevalent phyla were Rotifera, Arthropoda, and Chordata, with Keratella, Brachionus, and Macrothrix being notable taxa. In conclusion, zooplankton are vital components of aquatic ecosystems, and their diversity is crucial for ecological understanding and conservation efforts.

## Conclusion

Our study concluded that the COI gene marker sequencing provided the substantial information on zooplankton diversity of Dianchi Lake. The Caohai part had lowest diversity, highest COD and lowest transparency values indicating more pollution compared to other sampling sites. Further, the environmental parameters COD and transparency had great influence on zooplankton diversity especially Rotifera community. This study underscores the importance of preserving the biodiversity of Dianchi Lake’s zooplankton communities, particularly by controlling COD and maintaining water transparency to sustain its ecological resources and economic significance. However, further studies involving high throughput metagenomic sequencing are warranted to detect species within all taxonomic groups of zooplankton and elucidate the individual role of zooplankton species in aquatic ecosystems which is essentially required for ecosystem monitoring of zooplankton diversity.

## Data availability statement

The original contributions presented in the study are included in this article/supplementary material, further inquiries can be directed to the corresponding author.

## Ethics statement

The manuscript presents research on animals that do not require ethical approval for their study.

## Author contributions

FC: Conceptualization, Data curation, Formal analysis, Investigation, Methodology, Software, Validation, Visualization, Writing–original draft. SX: Data curation, Formal analysis, Investigation, Methodology, Software, Validation, Writing–review & editing. GY: Conceptualization, Formal analysis, Software, Validation, Writing–review & editing. MD: Conceptualization, Funding acquisition, Investigation, Project administration, Resources, Supervision, Validation, Writing–review & editing.

## References

[B1] Abe Y. Matsuno K. Fujiwara A. Yamaguchi A. (2020). Review of spatial and inter-annual changes in the zooplankton community structure in the western Arctic Ocean during summers of 2008–2017. *Prog. Oceanogr.* 186:102391.

[B2] Amorim C. A. Valença C. R. de Moura-Falcão R. H. do Nascimento Moura A. (2019). Seasonal variations of morpho-functional phytoplankton groups influence the top-down control of a cladoceran in a tropical hypereutrophic lake. *Aquatic Ecol.* 53 453–464.

[B3] Berry T. E. Saunders B. J. Coghlan M. L. Stat M. Jarman S. Richardson A. J. (2019). Marine environmental DNA biomonitoring reveals seasonal patterns in biodiversity and identifies ecosystem responses to anomalous climatic events. *PLoS Genet.* 15 e1007943. 10.1371/journal.pgen.1007943 30735490 PMC6368286

[B4] Bolger A. M. Lohse M. Usadel B. (2014). Trimmomatic: a flexible trimmer for Illumina sequence data. *Bioinformatics* 30 2114–2120. 24695404 10.1093/bioinformatics/btu170 PMC4103590

[B5] Bucklin A. Batta-Lona P. Questel J. Wiebe P. Richardson D. Copley N. (2022). COI metabarcoding of zooplankton species diversity for time-series monitoring of the NW Atlantic continental shelf. *Front. Mar. Sci.* 9:867893. 10.3389/fmars.2022.867893

[B6] Bucklin A. Peijnenburg T. C. Kosobokova K. N. O’Brien T. D. Blanco-Bercial L. Cornils A. (2021). Toward a global reference database of COI barcodes for marine zooplankton. *Mar. Biol.* 168:78. 10.1007/s00227-021-03887-y

[B7] Carugati L. Corinaldesi C. Dell’Anno A. Danovaro R. (2015). Metagenetic tools for the census of marine meiofaunal biodiversity: An overview. *Mar. Genomics* 24 11–20. 10.1016/j.margen.2015.04.010 25957694

[B8] Cicala F. Arteaga M. C. Herzka S. Z. Hereu C. M. Jimenez-Rosenberg S. P. A. Saavedra-Flores A. (2022). Environmental conditions drive zooplankton community structure in the epipelagic oceanic water of the southern Gulf of Mexico: A molecular approach. *Mol. Ecol.* 31 546–561. 10.1111/mec.16251 34697853

[B9] Deiner K. Walser J.-C. Mächler E. Altermatt F. (2015). Choice of capture and extraction methods affect detection of freshwater biodiversity from environmental DNA. *Biol. Conserv.* 183 53–63.

[B10] Ding Y. Shan B. Zhao Y. (2015). Assessment of river habitat quality in the Hai River Basin, Northern China. *Int. J. Environ. Res. Public Health* 12 11699–11717. 10.3390/ijerph120911699 26393628 PMC4586701

[B11] Djurhuus A. Pitz K. Sawaya N. A. Rojas-Márquez J. Michaud B. Montes E. (2018). Evaluation of marine zooplankton community structure through environmental DNA metabarcoding. *Limnol. Oceanogr.* 16 209–221. 10.1002/lom3.10237 29937700 PMC5993268

[B12] Dudgeon D. Arthington A. H. Gessner M. O. Kawabata Z.-I. Knowler D. J. Lévêque C. (2006). Freshwater biodiversity: importance, threats, status and conservation challenges. *Biol. Rev.* 81 163–182. 16336747 10.1017/S1464793105006950

[B13] Edgar R. C. (2013). UPARSE: Highly accurate OTU sequences from microbial amplicon reads. *Nat. Methods* 10, 996–998. 23955772 10.1038/nmeth.2604

[B14] Edgar R. C. Haas B. J. Clemente J. C. Quince C. Knight R. (2011). UCHIME improves sensitivity and speed of chimera detection. *Bioinformatics* 27 2194–2200. 10.1093/bioinformatics/btr381 21700674 PMC3150044

[B15] Everaert G. Deschutter Y. De Troch M. Janssen C. R. De Schamphelaere K. (2018). Multimodel inference to quantify the relative importance of abiotic factors in the population dynamics of marine zooplankton. *J. Mar. Syst.* 181 91–98.

[B16] Fabrin T. M. C. Stabile B. H. M. da Silva M. V. Jati S. Rodrigues L. de Oliveira A. V. (2020). Cyanobacteria in an urban lake: hidden diversity revealed by metabarcoding. *Aquatic Ecol.* 54 671–675.

[B17] Feng Q. Wang S. Liu X. Liu Y. (2020). Seasonal and spatial variations of phytoplankton communities and correlations with environmental factors in Lake Dianchi. *Beijing Da Xue Xue Bao* 56 184–192.

[B18] Fenoglio M. S. Rossetti M. R. Videla M. (2020). Negative effects of urbanization on terrestrial arthropod communities: A meta-analysis. *Glob. Ecol. Biogeogr.* 29 1412–1429.

[B19] Friedland K. D. Morse R. E. Shackell N. Tam J. C. Morano J. L. Moisan J. R. (2020). Changing physical conditions and lower and upper trophic level responses on the US Northeast Shelf. *Front. Mar. Sci.* 7:567445. 10.3389/fmars.2020.567445

[B20] Geller J. Meyer C. Parker M. Hawk H. (2013). Redesign of PCR primers for mitochondrial cytochrome c oxidase subunit I for marine invertebrates and application in all-taxa biotic surveys. *Mol. Ecol. Resour.* 13 851–861. 10.1111/1755-0998.12138 23848937

[B21] Haberman J. Haldna M. (2014). Indices of zooplankton community as valuable tools in assessing the trophic state and water quality of eutrophic lakes: long term study of Lake Võrtsjärv. *J. Limnol.* 73 263–273.

[B22] He L. Zhang L. Yang J. Zhao Z. Zhou X. Feng Q. (2021). Monitoring of plankton diversity in Dianchi Lake by environmental DNA technology. *IOP Conf. Ser. Earth Environ. Sci.* 651:42023. 10.13227/j.hjkx.202007236 33742874

[B23] Jo J. Oh J. Park C. (2020). Microbial community analysis using high-throughput sequencing technology: a beginner’s guide for microbiologists. *J. Microbiol.* 58 176–192. 10.1007/s12275-020-9525-5 32108314

[B24] Kostopoulou V. Carmona M. J. Divanach P. (2012). The rotifer *Brachionus plicatilis*: an emerging bio-tool for numerous applications. *J. Biol. Res.* 17:97.

[B25] Lee S.-R. Song E. H. Lee T. (2018). Eukaryotic plankton species diversity in the Western Channel of the Korea Strait using 18S rDNA sequences and its implications for water masses. *Ocean Sci. J.* 53 119–132.

[B26] Leray M. Yang J. Y. Meyer C. P. Mills S. C. Agudelo N. Ranwez V. (2013). A new versatile primer set targeting a short fragment of the mitochondrial COI region for metabarcoding metazoan diversity: application for characterizing coral reef fish gut contents. *Front. Zool.* 10:34. 10.1186/1742-9994-10-34 23767809 PMC3686579

[B27] Li G. B. Li L. Pan M. Xie Z. C. Li Z. X. Xiao B. D. (2014). The degradation cause and pattern characteristics of Lake Dianchi ecosystem and new restoration strategy of ecoregion and step-by-step implementation. *J. Lake Sci.* 26 485–496.

[B28] Li R. Y. Yang H. Zhou Z.-G. Lue J.-J. Shao X. H. Jin F. (2007). Fractionation of heavy metals in sediments from Dianchi Lake, China. *Pedosphere* 17 265–272. 10.1016/j.ecoenv.2020.110346 32120176

[B29] Lv J. Lin Y. Zhao Z. Zhou X. (2023). eDNA metabarcoding revealed the seasonal and spatial variation of phytoplankton functional groups in the Chai river and their relationship with environmental factors. *J. Freshw. Ecol.* 38:2176374.

[B30] Magoč T. Salzberg S. L. (2011). FLASH: fast length adjustment of short reads to improve genome assemblies. *Bioinformatics* 27 2957–2963. 10.1093/bioinformatics/btr507 21903629 PMC3198573

[B31] Martin M. (2011). Cutadapt removes adapter sequences from high-throughput sequencing reads. *EMBnet. J.* 17 10–12. 10.1089/cmb.2017.0096 28715235

[B32] Mimouni E.-A. Pinel-Alloul B. Beisner B. E. (2015). Assessing aquatic biodiversity of zooplankton communities in an urban landscape. *Urban Ecosyst.* 18 1353–1372.

[B33] Mwagona P. C. Chengxue M. Hongxian Y. (2018). Seasonal dynamics of Zooplankton functional groups in relation to environmental variables in Xiquanyan Reservoir, Northeast China. *Ann. Limnol.-Int. J. Limnol.* 54:33.

[B34] Nogueira M. G. (2001). Zooplankton composition, dominance and abundance as indicators of environmental compartmentalization in Jurumirim Reservoir (Paranapanema River), São Paulo, Brazil. *Hydrobiologia* 455 1–18.

[B35] Oksanen J. Blanchet F. G. Friendly M. Kindt R. Legendre P. McGlinn D. (2019). *Package ‘vegan.’ Community Ecology Package.* Vienna: The R Project for Statistical Computing.

[B36] Pineda A. Peláez O. Dias J. D. Segovia B. T. Bonecker C. C. Velho L. F. M. (2019). The El Niño Southern Oscillation (ENSO) is the main source of variation for the gamma diversity of plankton communities in subtropical shallow lakes. *Aquatic Sciences* 81 1–15. 17153703

[B37] Porter T. M. Hajibabaei M. (2018). Over 2.5 Million COI Sequences in GenBank and Growing. *PLoS One* 13:e0200177. 10.1371/journal.pone.0200177 30192752 PMC6128447

[B38] Qiong Y. Zhenyao L. I. U. Jidong Y. (2009). Simultaneous determination of chemical oxygen demand (COD) and biological oxygen demand (BOD5) in wastewater by near-infrared spectrometry. *J. Water Resour. Prot.* 4:2009.

[B39] Ratnasingham S. Hebert P. D. N. (2007). BOLD: The Barcode of Life Data System (http://www.barcodinglife.org). *Mol. Ecol. Notes* 7 355–364. 18784790 10.1111/j.1471-8286.2007.01678.x PMC1890991

[B40] Sarang T. Manoj K. (2021). Assessment of water quality of Tapi river with reference to rotifera community. *J. Entomol. Zool. Stud.* 9 2281–2284.

[B41] Schroeder A. Pallavicini A. Edomi P. Pansera M. Camatti E. (2021). Suitability of a dual COI marker for marine zooplankton DNA metabarcoding. *Mar. Environ. Res.* 170:105444. 10.1016/j.marenvres.2021.105444 34399186

[B42] Sherman K. Kane J. Murawski S. Overholtz W. Solow A. (2002). “The US Northeast shelf large marine ecosystem: Zooplankton trends in fish biomass recovery,” in *Large marine ecosystems* (Amsterdam: Elsevier), 195–215.

[B43] State Environmental Protection Administration (2002). *Water and wastewater monitoring and analysis method*, 4th Edn. Beijing: China Environmental Science Press.

[B44] Steinberg D. K. Landry M. R. (2017). Zooplankton and the ocean carbon cycle. *Annu. Rev. Mar. Sci.* 9 413–444. 10.1146/annurev-marine-010814-015924 27814033

[B45] Trebitz A. S. Hoffman J. C. Darling J. A. Pilgrim E. M. Kelly J. R. Brown E. A. (2017). Early detection monitoring for aquatic non-indigenous species: Optimizing surveillance, incorporating advanced technologies, and identifying research needs. *J. Environ. Manage.* 202 299–310. 10.1016/j.jenvman.2017.07.045 28738203 PMC5927374

[B46] Vaughn C. C. (2010). Biodiversity losses and ecosystem function in freshwaters: emerging conclusions and research directions. *BioScience* 60 25–35.

[B47] Vörösmarty C. J. McIntyre P. B. Gessner M. O. Dudgeon D. Prusevich A. Green P. (2010). Global threats to human water security and river biodiversity. *Nature* 467 555–561. 20882010 10.1038/nature09440

[B48] Walkusz W. Kwasniewski S. Petersen S. F. Hop H. Tverberg V. Wieczorek P. (2009). Seasonal and spatial changes in the zooplankton community of Kongsfjorden, Svalbard. *Polar Res.* 28 254–281.

[B49] Wang J. He L. Yang C. Dao G. Du J. Han Y. (2018). Comparison of algal bloom related meteorological and water quality factors and algal bloom conditions among Lakes Taihu, Chaohu, and Dianchi (1981–2015). *J. Lake Sci* 30 897–906.

[B50] Wang J.-H. Wang Y.-N. Dao G.-H. Du J.-S. Han Y.-P. Hu H.-Y. (2020). Decade-long meteorological and water quality dynamics of northern Lake Dianchi and recommendations on algal bloom mitigation via key influencing factors identification. *Ecol. Indic.* 115:106425.

[B51] Wang M. Wang Y. Duan L. Liu X. Jia H. Zheng B. (2022). Estimating the pollutant loss rate based on the concentration process and landscape unit interactions: a case study of the Dianchi Lake Basin, Yunnan Province, China. *Environ. Sci. Pollut. Res.* 29 77927–77944. 10.1007/s11356-022-19696-9 35688977

[B52] Wang W. Sun S. Sun X. Zhang G. Zhang F. (2020). Spatial patterns of zooplankton size structure in relation to environmental factors in Jiaozhou Bay, South Yellow Sea. *Mar. Pollut. Bull.* 150:110698. 10.1016/j.marpolbul.2019.110698 31744604

[B53] Wang Y. Wang W. Wang Z. Li G. Liu Y. (2018). Regime shift in Lake Dianchi (China) during the last 50 years. *J. Oceanol. Limnol.* 36 1075–1090.

[B54] Wang Z. Xiao B. Wu X. Tu X. Wang Y. Sun X. (2010). Linear alkylbenzene sulfonate (LAS) in water of Lake Dianchi—spatial and seasonal variation, and kinetics of biodegradation. *Environ. Monit. Assess.* 171 501–512. 10.1007/s10661-009-1295-9 20072810

[B55] Wang Z. Zhang Z. Zhang J. Zhang Y. Liu H. Yan S. (2012). Large-scale utilization of water hyacinth for nutrient removal in Lake Dianchi in China: the effects on the water quality, macrozoobenthos and zooplankton. *Chemosphere* 89 1255–1261. 10.1016/j.chemosphere.2012.08.001 22939513

[B56] Xiong W. Huang X. Chen Y. Fu R. Du X. Chen X. (2020). Zooplankton biodiversity monitoring in polluted freshwater ecosystems: A technical review. *Environ. Sci. Ecotechnol.* 1:100008.

[B57] Xiong W. Ni P. Chen Y. Gao Y. Li S. Zhan A. (2019). Biological consequences of environmental pollution in running water ecosystems: A case study in zooplankton. *Environ. Pollut.* 252 1483–1490. 10.1016/j.envpol.2019.06.055 31265959

[B58] Yang J. Zhang X. (2020). eDNA metabarcoding in zooplankton improves the ecological status assessment of aquatic ecosystems. *Environ. Int.* 134:105230. 10.1016/j.envint.2019.105230 31704569

[B59] Yang J. Zhang X. Xie Y. Song C. Sun J. Zhang Y. (2017). Ecogenomics of zooplankton community reveals ecological threshold of ammonia nitrogen. *Environ. Sci. Technol.* 51 3057–3064. 10.1021/acs.est.6b05606 28191935

[B60] Zhao L. Zhang X. Xu M. Mao Y. Huang Y. (2021). DNA metabarcoding of zooplankton communities: species diversity and seasonal variation revealed by 18S rRNA and COI. *PeerJ* 9:e11057. 10.7717/peerj.11057 33777533 PMC7983862

